# Editorial: “Effective strategies for promoting health-enhancing children's physical activity”

**DOI:** 10.3389/fpubh.2022.964316

**Published:** 2022-07-26

**Authors:** Radenko M. Matic, Ivana M. Milovanović, Irena Valantine, Kostas Alexandris, Stevo Popovic

**Affiliations:** ^1^Faculty of Sport and Physical Education, University of Novi Sad, Novi Sad, Serbia; ^2^Western Balkan Sport Innovation Lab, Podgorica, Montenegro; ^3^Lithuanian Sports University, Kaunas, Lithuania; ^4^Aristotle University of Thessaloniki, Thessaloniki, Greece; ^5^Faculty for Sport and Physical Education, University of Montenegro, Niksic, Montenegro

**Keywords:** active lifestyle, exercise, children, health behavior, interventions

## Introduction

The issue of low children's physical activity (PA) has been important research and professional topic for years (and decades). Its importance increased after the restrictive measures caused by the COVID-19 pandemic (1). PA of children is crucial to the not only physical, but also psychological, social, and cognitive health of children (2, 3). Baring this in mind, there are several gudelines and protocols which recommend that children and youth spend a minimum of 60 min each day in moderate- to vigorous-intensity PA [e.g., (4, 5)]. Researchers tried to explain factors that contribute to modifying behavior (in this context, health behavior) and factors that contribute to formulation of different conceptual models (Bandura's social learning model, Rogers' Innovation Diffusion Theory, etc.) Contemporary society needs a particular scientific focus on the issue of establishment of effective strategies for promoting health-enhancing physical activity for children (6). Explanation of the mechanism of the impact of children's PA and socially appropriate behavior on health is essential for improvement of social interventions strategy. Effective strategies for improving childrens PA and adapting an active lifestyle reduce the spread of mass non-communicable diseases and children's antisocial behavior. Therefore, adopted patterns of children's active behavior can impact their habits over the upcoming years (7).

The topic provides high-quality research on promotion of health-enhancing children's PA, which can offer guidelines for researchers and policymakers at local, regional, national, and international levels. These guidelines can contribute to social intervention by encouraging children's PA with the active role of a certified institution (families, schools, sports organizations, and local communities).

## Contribution to the field

This Research Topic has been proposed due to challenges for improvement of children's socially relevant health behavior. It aims to identify existing changes in behavior relevant to health, where PA is an important factor for the change of children's lifestyle. The editorial aimed to scientifically present strategies associated with health-enhancing children's PA and to choose intervention methods for promotion of children's healthy lifestyles. The purpose of the Research Topic was to contribute to cutting-edge knowledge in explaining effective strategies for promoting health-enhancing children's PA. The outcome of this Research Topic is 18 published articles which contribute to the mentioned field from different research aspects.

General insight into the Research Topic shows that the authors of the published manuscripts paid attention to various effective strategies that increase children's PA related to health: (1) active transport to school, (2) family interventions, (3) interventions in school settings and inclusive school environment, (4) academic performance, (5) impact of the geographical and living environment, (6) effectiveness of adequate sport or recreational programs, (7) motor coordination programs improvement, (8) sedentary patterns and health-related physical fitness, and (9) mediator role of sleep.

Firstly, Huang et al. presented valuable findings on the association between active travel to school (ATS) and PA and screen time by individual and parental characteristics among Chinese adolescents. The authors concluded that ATS is a helpful approach in promotion of PA, determined by personal (gender, age, living environment) and parental characteristics (parental educational level and occupations). Yesiltepe et al. considered that cycling, as a way of active transport opportunity, is an individual and social contribution to public health, environmental protection, and climate change control. Therefore, current pandemic conditions could contribute to faster acceptance of cycling as a win-win strategy for individuals, families, and communities. Continuation of that segment of the green agenda and adequately responding to pandemic issues could be children's using outdoor space as an effective solution for PA practice, especially in urban environments proposed by Ma et al. Additionally, the research conducted by Planinšec et al. emphasized that researchers and decision-makers should create and develop an effective strategy for promotion of health-enhancing children's PA in case of similar situations or future lockdown.

Huang et al. conducted a meta-analysis about family interventions to PA and sedentary behavior. Authors included studies published in 2012 and later. They considered the role model of family members to their children and saw family interventions as a promising way of promoting children's PA. Additionally, a systematic review examined PA levels of children and adolescents with or without autism spectrum disorder (ASD) in inclusive schools by Li et al. Liang et al. emphasized the mediating role of sleep between PA and executive function in children with attention to deficit hyperactivity disorder (ADHD), as an essential factor of effective strategies in children's PA Therefore, regular sleep patterns represent a quality base for human health and maintenance of homeostatic needs.

Furthermore, two studies confirmed the significant influence of children's PA on increase of their academic performance (Durić et al.). A systematic review by Petrigna et al. identified that the time spent in PA could encourage their cognitive development and indirectly impact improving academic performance and motor competencies. Learning through movement is suggested as an effective, low-cost, and enjoyable strategy for elementary schoolchildren. On another side, one study (Aleksić Veljković et al.) showed that children's participation in yoga intervention programs increases their motor skills but not their cognitive abilities.

Additionally, when it comes to improvement of motor competencies and health-enhancing children's PA, some studies revealed potential programs that should consist of directions related to gender (Zhang et al.), as well as age and gender (Battaglia et al.), living setting, geographical area, and gender (Gallotta et al.). Progression in physical fitness could be expected with the addition of exercise sessions more than the regular PA curriculum. Therefore, the physical fitness of adolescent girls can be improved with additional school interventions (on the top of regular school physical education activities) by Petrušič et al.

However, the main concern and potential problems are shown in research by Giuriato et al. Namely, authors speculate that nowadays, young people achieve fewer motor experiences than peers in the past, with geographical and sociocultural determinants. Consequently, decreasing sedentary time and promoting physical benefits during sedentary breaks sounds like a possible effective strategy in changing sedentary time patterns proposed by Lu et al. The research conducted by Al-Daghri et al. is compatible with previously mentioned research. It showed that childhood obesity and pediatric metabolic syndrome (MetS) have steadily increased during the last decade in Saudi Arabia. The authors presented results of intervention programs to prevent cardiometabolic disorders in Arab youth and reported that those programs had modest effects due to COVID-19 imposed lockdowns. Finally, research designed by Feng et al. presents a study protocol article with an in-depth description of the intervention, which is based on a three-arm randomized controlled trial, which will comprise a 12-week intervention and a 12-week follow-up. Authors expect that the proposed study will improve preschoolers' movement behaviors and health outcomes, as well as their parents' movement behaviors.

## Conclusion

This Research Topic aimed to identify the effective strategies and good practices for promoting health-enhancing physical activity in childhood. This is due to the fact, that society needs an adequate strategy in maintaining an increasingly active lifestyle for children. This type of societal intervention requires support from institutions from different levels: from the individual (home, family level) to sports organizations (group level) to and finally to the local communities (societal level).The various articles put out on this research topic indicate the complexity of children's activation in physical activity in contemporary society. Implementation of suggested strategies for promoting health-enhancing children's PA is determined by using the comprehensive knowledge and its implementation in family, school settings, sports organizations, and local environments. It can be further developed in different contexts to adapt to conditions that must be fulfilled. It seems that more effective strategies nowadays than ever require the inclusion of more community campaigns, improving access to physical activity infrastructure, and involving more sectors.

## Author contributions

RM drafted the editorial. SP, IM, IV, and KA revised and approved the final version. All authors contributed to the article and approved the submitted version.

## Funding

This editorial has been part of the Faculty of Sport and Physical Education project (Reg. No: 142-451-2596/2021) that were financed by the Provincial Secretariat for Higher Education and Scientific Research and also the editors gratefully acknowledge above mentioned secretariat.

## Conflict of interest

The authors declare that the research was conducted without any commercial or financial relationships that could be construed as a potential conflict of interest.

## Publisher's note

All claims expressed in this article are solely those of the authors and do not necessarily represent those of their affiliated organizations, or those of the publisher, the editors and the reviewers. Any product that may be evaluated in this article, or claim that may be made by its manufacturer, is not guaranteed or endorsed by the publisher.
